# Elevated Membrane and Soluble CD64: A Novel Marker Reflecting Altered FcγR Function and Disease in Early Rheumatoid Arthritis That Can Be Regulated by Anti-Rheumatic Treatment

**DOI:** 10.1371/journal.pone.0137474

**Published:** 2015-09-25

**Authors:** Peter Matt, Ulla Lindqvist, Sandra Kleinau

**Affiliations:** 1 Department of Medical Sciences, Uppsala University, Uppsala, Sweden; 2 Department of Cell and Molecular Biology, Uppsala University, Uppsala, Sweden; Nippon Medical School Graduate School of Medicine, JAPAN

## Abstract

**Objectives:**

Fc receptors (FcR) interacting with immune complexes (ICs) is a central event in the immune pathogenesis of rheumatoid arthritis (RA). Here we asked if a specific FcR is linked to RA pathogenesis and if FcR activities relate to disease and treatment outcome in early RA.

**Material and Methods:**

Twenty autoantibody-positive RA patients and 33 HC were included. The patients were evaluated before and after treatment with methotrexate and prednisolone. At follow-up, the EULAR response criteria were applied to determine the individual treatment outcomes. Serum immunoglobulin levels were measured and the expression of FcR for IgG (FcγR) and IgA (FcαR) on peripheral blood monocytes were determined by flow cytometry. The monocytic FcγR function was evaluated by human IgG1 and IgG3 IC-binding and TNFα stimulated release. Plasma levels of soluble FcRs (sFcRs) were determined with ELISA.

**Results:**

The IgG1 and IgG3 levels were elevated in the RA sera. The RA monocytes expressed more CD64 and cell surface-bound IgG than HC monocytes, and showed an impaired FcγR function as reflected by changes in IC-binding and decreased IC-stimulated TNFα secretion. These findings correlated significantly with different disease activity markers. Furthermore, sFcRs were elevated in the patient plasma, and sCD64 was specific for RA (compared with a reference group of patients with active psoriatic arthritis). Following treatment, immunoglobulins and sFcR levels were reduced, whereas membrane CD64 was only decreased in patients with good response to treatment.

**Conclusions:**

Early RA patients display increased membrane and soluble CD64 and an impaired FcγR function correlating with joint disease activity. Beneficial responses of anti-rheumatic treatment in patients reduce CD64. These data suggest sCD64 as an important objective biomarker in RA.

## Introduction

Rheumatoid arthritis (RA) is a chronic inflammatory, joint-destructive disease in which antibodies (abs) to self-antigens are believed to contribute to the pathogenesis. It is known that healthy individuals with elevated serum levels of IgG and IgA are at increased risk of developing RA later in life. In established RA, high levels of immunoglobulins (Igs) are evident in serum and joint fluid, and the synovial membrane offers no barrier for Igs and immune complexes (ICs) to diffuse between these two compartments [1–7]. Hence, a disturbed IC handling could play a crucial role for initiating and perpetuating the disease [8]. Among the many known auto-abs reported in RA, anti-cyclic citrullinated peptide abs (ACPAs) and rheumatoid factors (RFs) have become the gold standard for RA diagnosis, and high titres of these are associated with a more aggressive disease [9,10].

Monomeric Ig or Ig in ICs initiates immunological and inflammatory reactions by interacting with Ig-specific membrane-bound Fc receptors (FcR) on immune competent cells, including monocytes and joint-stationed macrophages [11–13]. The human IgG FcR family includes FcγRI (CD64), FcγRII (CD32) and FcγRIII (CD16) [8]. In addition, FcγRII exists in the three isoforms a, b and c, while FcγRIII has two isoforms, a and b. All FcγRs are activating receptors except FcγRIIb which is an inhibitory receptor. Monocytes express CD64, CD32a,b,c and CD16a, as well as the FcR for IgA (FcαR; CD89)[14]. The latter receptor can either stimulate or inhibit inflammatory processes [15,16]. In an FcR-mediated immune response not only the receptor type is of importance, also the FcR's binding affinity for its corresponding Ig is central. Thus, changes in the Ig glycosylation state and polymorphisms in the FcR ligand-binding domain may improve or weaken the interaction between the Ig/or IC with its corresponding FcR [17]. Most of the FcγRs are low-affinity receptors and will only bind IgG in ICs. However, CD64 is the only high-affinity receptor that can bind monomeric IgG in addition to the binding of polymeric IgG of all subclasses, except for IgG2 [18]. While cell activation by low-affinity receptors requires crosslinking of at least two FcRs by ICs, one single CD64 prebound with monomeric IgG can fulfill the same task. It can therefore be assumed that the CD64 status of a cell can influence IC handling and IC-mediated inflammation. Interestingly, synovial tissue of healthy individuals expresses CD16 and CD32 but not CD64, whereas CD64 is strongly expressed in RA synovium [19]. Furthermore, the pathogenicity of this high affinity FcγR has been confirmed in animal studies; CD64- or FcRγ-chain deficient mice demonstrated decreased arthritic symptoms in the collagen-induced arthritis model, and treatment with a CD64-directed immunotoxin showed promising results in human CD64 transgenic rats suffering from joint inflammation [20–22].

Previous monocyte FcγR expression studies in RA have reported conflicting findings, showing increased, decreased or similar expressions of CD64, CD32a or CD16a compared with HC [23–27]. The reasons for these diverging outcomes are probably due to differences in the patients`disease duration, ongoing treatments, auto-ab statuses, and in the age- and sex matching of patients and controls. Thus, the expression level of an FcR reflects its total turnover (i.e. receptor uptake, secretion, shedding and re-expression), and such expression levels are known to be influenced by pro- and anti-inflammatory cytokines, hormones, age and medication [28–36].

Interestingly, the FcRs also act as binding sites for acute-phase proteins such as serum amyloid A (SAP) and C-reactive protein (CRP) [37–39]. It is believed that the acute-phase proteins generate pro-inflammatory cytokines in this manner, thus strengthening the inflammatory response. CRP is a common marker in blood for disease activity determination in RA, but CRP can also be detected in the rheumatoid synovium [40, 41].

We have recently shown that the FcγR expression on monocytes/macrophages is modified in established RA and that patients with an active disease have a dysregulated FcγR function despite ongoing anti-rheumatic treatment [42]. In order to extend our knowledge of monocyte FcR activity in the early phase of RA, we conducted a study on drug-naïve, auto-ab-positive early RA patients. We determined the monocyte FcR status and soluble FcRs in plasma before and after anti-rheumatic treatment and asked if a specific FcR activity is linked to RA pathogenesis and possibly predict a response to anti-rheumatic treatment, and finally if there are specific FcRs that objectify RA disease activity.

## Material and Methods

### Subjects

Twenty newly diagnosed early RA patients and 33 age and sex matched healthy blood donors (HC) visiting the Rheumatology Department or the Blood Donor Clinic at the University Hospital in Uppsala were studied (**Table 1**).

**Table 1:** Characteristics of healthy controls and early RA patients.

*Parameters*	*Healthy controls (blood donors)*	*Early naive rheumatoid arthritis patients*
**Total numbers**	33	20
**Gender (M;F)**	17;16	8;12
**Median age, years**	53.0 (21–66)	53.5 (19–77)
**Mean disease duration, months**	n.a.	8.2
**Mean DAS28**	n.a.	5.26 (3.16–7.10)
**Mean DAS28-CRP**	n.a.	4.92 (2.44–6.93)
**Mean HAQ-score**	n.a.	0.95 (0.25–2.00)
**Mean E-SR mm/h**	n.d.	28.3 (5–68)
**Mean CRP mg/L**	n.d.	26.0 (0.46–177)
**Number of RF⁺**	0	19
**Mean titre IU/mL**	-	287.4 (<20–1550)
**Number of ACPA IgG⁺**	0	20
**Mean titre U/L**	-	1153 (9–8480)

Ranges are presented in brackets. DAS28 = disease activity score (28 joint index), HAQ = health assessment questionnaire, E-SR = sedimentation rate, CRP = C-reactive protein, RF = rheumatoid factor, ACPA = anti–citrullinated protein antibody, n.a. = not applicable, n.d. = not done.

Written informed consent according to the Helsinki Declaration was obtained from all participants, and the local ethics committee at Uppsala University approved the study. All patients fulfilled the 2010ACR/Eular classification criteria of RA [43]. Joint evaluations were performed by an experienced rheumatologist and the current disease activity was determined using an online DAS28/DAS28-CRP calculator. Ongoing infectious disease was considered an exclusion criterion. The patients assessed their disease activity with the HAQ (Health Assessment Questionnaire) form and the VAS (Visual Analogue Scale) scales (reference: 0–100mm) for morning stiffness (MS), global health (PG) and pain. The MS values were calculated as the mean results of questions 5 and 6 of the BASDAI (Bath ankylosing spondylitis disease activity index). A mean HAQ of 0.95 (range 0.25–2.00), a mean VAS of MS of 60.5mm (range 21–95mm), a mean PG of 52.4mm (range 8–96mm) and a mean pain VAS of 60.9mm (range 27–96mm) were obtained.

Analyses for Ig isotypes, IgG subclasses and auto-abs were performed at the departments of Clinical Chemistry and Clinical Immunology at Uppsala University Hospital using standardized methods; RFs and ACPA were detected with Phadia EliA (ThermoScientific, USA). Auto-ab positive patients were selected in order to evaluate patients with initially negative prognostic factors. Eight patients had detectable low titres of anti-nuclear abs (ANA) of either homogeneous or speckled pattern. These patients did not fulfill the criteria of any other systemic inflammatory disease.

The patients were evaluated twice: at the first visit (FV) and at a follow-up (FU) visit after 3–4 months of treatment. Initially all patients were steroid and DMARD naïve. Unless contra-indicated, per oral treatment with methotrexate (MTX) and prednisolone (PRED) was initiated after FV (as recommended by the Swedish national guidelines). A total of 17 patients were given MTX (mean weekly dose of 15.2 mg at FU), 2 patients received treatment with sulfasalazine (mean daily dose of 2000 mg at FU) and one patient was treated with anti-malarial (mean daily dose of 160 mg chloroquine phosphate at FU). Seventeen patients received PRED (mean daily dose of 7.9 mg at FU), the remaining 3 patients were treated with intra-articular steroid injections at FV. At FU the treatment effect was evaluated with the EULAR response criteria for RA [44]; 7 patients were identified as good, 5 as moderate and 8 as non-responders. All good responders had received MTX + PRED, among the non-responders this proportion was 62.5%. Good responder data were later compared with the non-responder data.

### Cell isolation

Daily fresh EDTA-treated venous blood was collected from patients and controls, and peripheral blood mononuclear cells (PBMCs) were isolated by Ficoll-Paque (GEHealthcare, Uppsala/Sweden). Monocytes were further separated from the PBMCs by magnetic activating cell sorting (MACS) using anti-CD14 microbeads and LS columns according to the Manufacturer's (MiltenyiBiotec, Germany) instructions. With this method the obtained monocyte purity is usually >95%.

### Abs and flow cytometry

The PBMCs were stained with the following phycoerythrin (PE)- or fluorescein isothiocyanate (FITC)-conjugated mouse anti-human abs: anti-CD14PE (clone 61D3; Biosite, Sweden), anti-CD14FITC (clone TÜK4; Dako, Denmark), anti-CD16a (PE- and FITC- conjugated clone DJ130c; Dako), anti-CD89PE (clone A59; BD Pharmingen, USA), anti-CD64FITC (clone 10.1; BioLegend, USA) and anti-CD32aFITC (clone AT10 (recognizing CD32a,b,c); Abdserotec, United Kingdom). Biotin-conjugated mouse anti-human IgG (clone G18-145; BD Pharmingen, USA) and streptavidin-labelled FITC or PE (BioLegend) were used to detect surface bound IgG on CD16⁺ or CD64⁺ cells. In addition, mouse anti-human CD32b (clone GB3 (recognizing CD32b,c); Suppremol, Germany) [19, 45] was used followed by PE-conjugated rabbit anti-mouse IgG (clone STAR12-A; Abdserotec). Isotype matched PE-conjugated mouse IgG1 (clone DAK-G01; Dako), FITC-conjugated mouse IgG1 (clone DAK-G01; Dako) and FITC-conjugated mouse IgG2a (clone DAK-G05; DakoCytomation, Denmark) served as controls for non-specific staining.

The ab-stained cells were analysed in a FACScan (serial nr 82121; BectonDickinson, USA) and the data processed with the FlowCellQuest software. The PBMC population was gated in a forward and side scatter diagram and the monocytes further defined by their CD14 expression. The quadrant markers were set based on isotype controls and negative populations. The frequency of positive cells was analysed in the PBMC gate. Individual monocyte FcR expressions are presented as mean fluorescence intensity (MFI).

### IC-binding by monocytes

The IC-binding assay was performed as previously described [42]. Briefly, human ICs were formed with RhD⁺ red blood cells (RBC) from a healthy blood donor and human IgG1 anti- RhD⁺ ab (clone BIRMA-D6) or human IgG3 anti- RhD⁺ ab (clone BRAD-3) (Bristol Institute For Transfusion Sciences, United Kingdom)[46]. RBC in phosphate buffered saline (PBS) was used as negative control. After IC formation, the samples were left on ice. One hundred μl of MACS-sorted monocytes (0.2x10^6^ cells in PBS) were mixed with 175 μl ICs or RBC (only in PBS), centrifuged at 1600 rpm/1 min and then incubated at 37°C for 30 minutes. After incubation the cell pellet was resuspended and put on ice. Next the cells were stained with ClayAdamsSedistain (Becton/Dickinson) and immediately examined; 45 μl of the monocyte-IC suspension was placed on a glass slide and at least 100 monocytes were counted in a light microscope at 400x magnification. A monocyte with 3 or more bound RBCs was defined as a rosette indicating IC binding. Signs of IC phagocytosis were not observed. Only samples allowing duplicate evaluation were taken into consideration and the mean percentage of rosettes was determined. All samples were 'blinded' towards the examiner. A binding ratio was also determined by dividing the percentage of IgG1 rosettes with the percentage of IgG3 rosettes. This ratio will represent the balance of the monocyte binding capacity of the two subclasses of ICs evaluated.

### Stimulation and detection of TNFα

MACS-sorted CD14 positive monocytes were stimulated with either kappa purified human IgG1 ab (clone BP078; BindingSite, USA) or IgG3 ab (clone BP082; BindingSite). As background control, F(ab)²-fragments of IgG (clone A50177H; Biodesign international, USA) was used. Lipopolysaccharide (LPS) or phorbol 12 myristate acetate (PMA) were used as positive controls. In 96 well microtiter plates (Maxisorp 442404; Thermo Scientific) abs or F(ab)²-fragments in a concentration of 1 μg ab/50 μl PBS per well were immobilized in triplicates over night at 4°c. On the next day the plates were washed 3 times in PBS and 1.3·10^6^ of monocytes (diluted in 200 μl serum free RPMI 1640) were added to each well. One μl LPS (2 μg/ml) was added to one triplicate set of positive control wells and 10 μl PMA (0.21 ng/ml) to another triplicate set of positive control wells. The plates were incubated in a humified CO²-chamber at 37°C for 20 h and on the next day the triplicates of supernatants were harvested, pooled and stored at -20°c for later analysis.

The supernatants were analyzed in an anti-TNFα ELISA as described [31, 42]. Briefly, microtiter plates were coated with mouse anti-human TNFα capture ab (2 μg/ml) (MAB610/clone 28401; R&D Systems, USA) overnight in a humid chamber. After washing and blocking with bovine serum albumin in PBS the plates were incubated for 2 h with supernatants or recombinant TNFα. After washing, biotinylated goat anti-human TNFα (300 ng/ml) (BAF210; R&D Systems) was added to the wells. Following washings, streptavidin-HRP or ExtrAvidin-peroxidase was added to the wells for 1h. After washing, the chromogenic substrate 1-step-Ultra-3,3`,5,5`tetramethylbenzidine (TMB) (Thermo Scientific) was added. The enzymatic reaction was stopped with 1 M H_2_SO_4_ and the plate finally read at 450 nm in a VERSA max microplate reader (Molecular Devices, USA). Data were analysed with the SoftMaxPro4.8 software. The results are presented as stimulation indices (i.e. IgG1 IC-stimulated TNFα-production divided with F(ab)²-stimulated TNFα-production). Such an index describes a factor of modified TNFα secretion in comparison with basal monocyte TNFα secretion.

### ELISA for soluble FcR

Concentrations (conc) of soluble (s) CD89, CD64 and CD16a in plasma were determined in patients and controls by commercial sandwich ELISA kits (for sCD89: cat no. CSB-EL008531HU and for sCD64: cat no. CSB-EL008537HU; Cusabio, P.R. China) (for sCD16a: cat no. SEB278Hu; Cloud-Clone Corp, USA) according to the Manufacturers' recommendations. For comparison, plasma samples from 20 age- and sex-matched psoriatic arthritis patients with active polyarticular disease (mean DAS28 = 4.1) were analyzed (the characteristics of these are presented in [47]). Briefly, microtiter plates pre-coated with anti-human CD89, CD64 or CD16a capture abs were incubated at 37°C for 2 h with 100 μl/well of standard (in a 2-fold dilution series) or thawed plasma samples in duplicate. After removal of the liquid, 100 μl of biotinylated anti-human CD89, CD64 or CD16a abs was added to each well, and the plates further incubated for 1 h. After washings, the plates were incubated with horseradish peroxidase-avidin (for 1h at 37°C), further washed and finally TMB substrate was added for another 30 min of incubation at 37°C. The color reaction was stopped with sulphuric acid (H_2_SO_4_) and the plates at last read in a VERSA max microplate reader. The obtained data were analysed with SoftMaxPro4.8 software. All individual results are presented as the mean of two independent samples.

### Statistical analyses

Graph Pad Prism software was used for statistical analyses. Mann Whitney U-test, Spearman Rank correlation and regression analyses were performed on the recommendation of a university statistician. Matched samples were compared using the Wilcoxon Signed-Rank Test. P-values < 0.05 were considered significant.

## Results

### Increased CD64 and cell surface-bound IgG on monocytes in early RA

**Fig 1:**
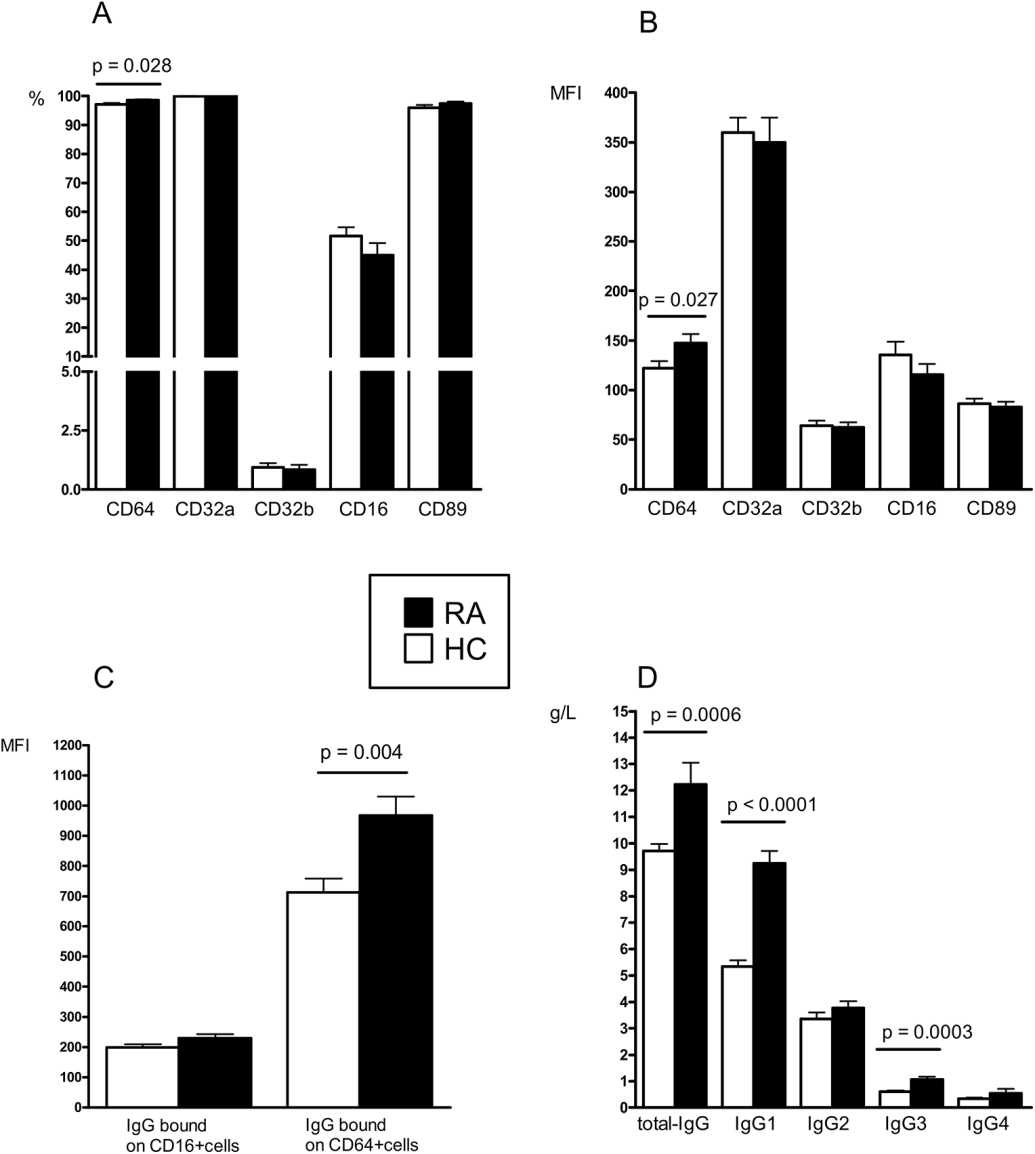
FcγR and Igs are increased in early RA. Expression of FcRs on monocytes (A-B), monocyte bound IgG (C) and IgG serum levels (D) in DMARD- and steroid naive early RA patients (*n* = 20) compared with healthy controls (HC) (*n* = 33). (MFI = mean fluorescence intensity).

Early RA patients presented at the FV an increase in the number of CD64-expressing monocytes and increased CD64 expression per monocyte (MFI) compared with HC (**Fig 1A and 1B**). When subdividing all cells in the PBMC gate into CD16 low, high and negative cells, all RA subsets had increased CD64 expression in comparison with HC subsets (**S1 Fig**). Expression (frequency and MFI) of CD32a, CD32b, CD16 and CD89 did not distinguish between patients and controls. The number of CD14 expressing cells (monocytes) was the same in both groups (data not shown). When investigating membrane-bound IgG on the monocytes, we detected an increase in IgG (MFI) on CD64⁺ cells in the RA patients, compared with the HC (**Fig 1C**). Regarding the amount of IgG bound on CD16⁺ cells, no difference was seen between the groups. In general however, CD16⁺ monocytes had bound less IgG than their CD64-expressing counterparts. This implies that in active disease CD64 is the main target for the binding of monomeric IgG on monocytes. Given that the RA monocytic IgG load was increased, we then compared the serum Ig levels in patients vs. controls. Signs of polyclonal B-cell activation were observed in the patients, and the total IgG and the opsonizing/complement activating IgG1 and IgG3 subclass levels were raised (**Fig 1D**). No differences regarding IgG2 and IgG4 concentrations were found between the groups. The observed mean IgG1 level (9.1 g/L) in the RA patients exceeded the normal reference range (2.8–8.0 g/L). However, the remaining IgG subclass levels and Ig isotype levels (IgM and IgA) were all within the reference ranges (data not shown).

### Monocyte FcR expressions correlate with disease activity markers in early RA

The increase in monocytic CD64 expression reflected the patient-reported disease activity (morning stiffness and pain), and the number of IgG-loaded CD64⁺ cells correlated positively with both DAS28-CRP and CRP (**Table 2**). From these data we conclude that monocyte CD64 up-regulation and increased monocyte IgG burden may be important early in the disease.

**Table 2:** Fcγ receptor expression and function reflecting severity of joint inflammation in drug naïve early RA patients (*n* = 20).

*FcγR related parameter*	*Correlation with*	*r/p*
**CD64 MFI**	MS	0.4648 / 0.004
	Pain-VAS	0.5064 / 0.023
**% CD64 positive cells with bound IgG**	DAS28-CRP	0.4806 / 0.032
	CRP	0.5859 / 0.007
**IgG1 IC binding**	TJC	- 0.6470 / 0.005
	DAS28-CRP	- 0.4623 / 0.062
**Ratio of IgG1: IgG3 IC binding**	SJC	- 0.5277 / 0.029
**IgG3 stimulated TNFα release**	PG	- 0.4975 / 0.042
**Soluble CD64**	CD64 MFI	0.4591 / 0.0417
	CD64 IgG MFI	0.5424 / 0.0135
**Soluble CD16A**	DAS28-CRP	0.8602 / < 0.0001
	E-SR	0.6682 / 0.0013
	SJC	0.8505 / < 0.0001
	HAQ	0.6753 / 0.0011
	Pain-VAS	0.5561 / 0.0109

Abbreviations; MFI = mean fluorescence intensity, MS = morning stiffness, VAS = visual analogue scale, DAS = disease activity score, CRP = C-reactive protein, IC = immune complex, TJC = tender joint count, SJC = swollen joint count, TNFα = tumor necrosis factor alpha, PG = patient global assessment of disease activity, E-SR = sedimentation rate, HAQ = health assessment questionnaire, r / p = regression coefficient / p-value.

### Monocyte Fcγ receptor function is altered in early RA

**Fig 2:**
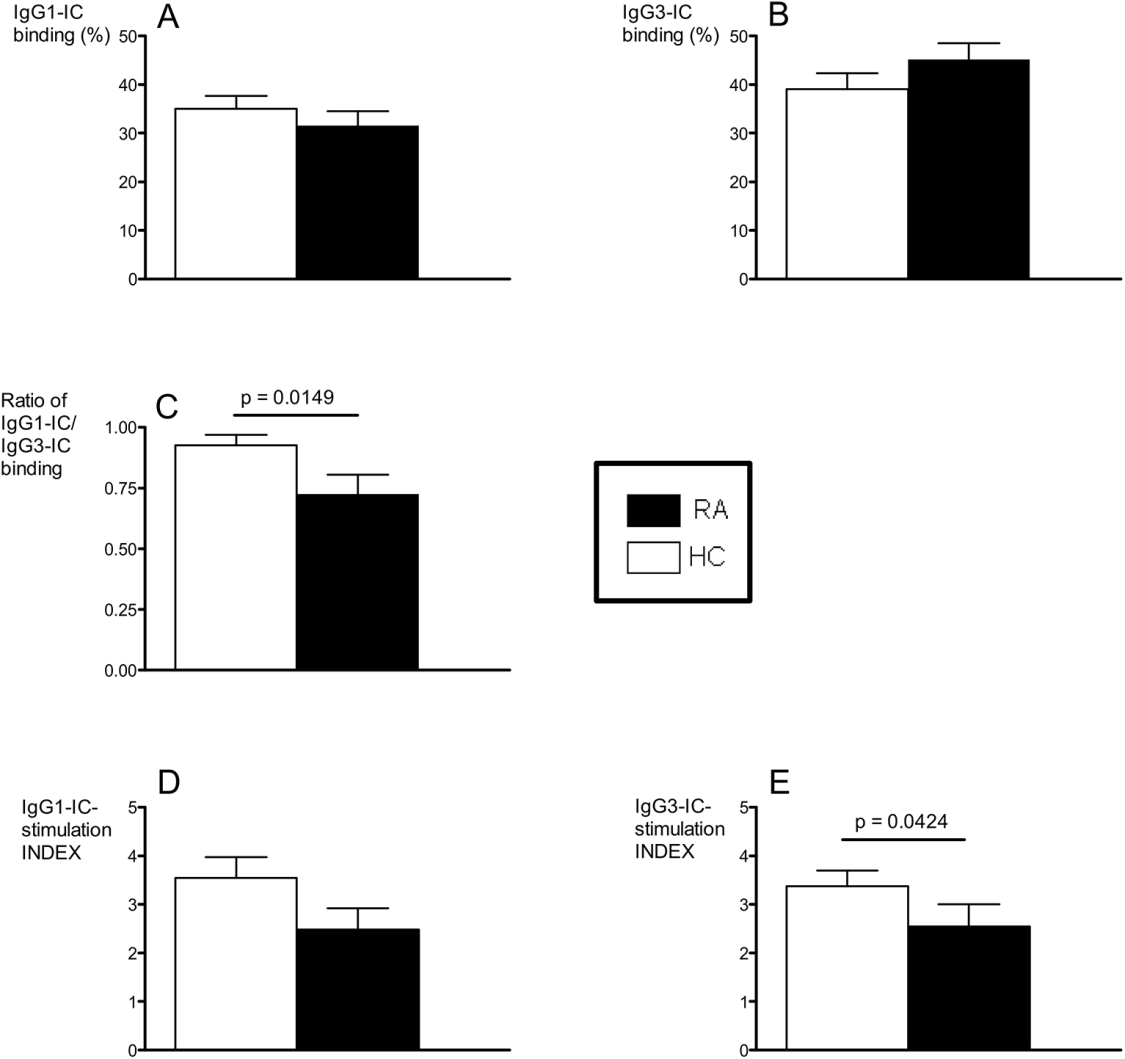
Altered FcγR function in early RA. IgG1 and IgG3 IC-binding (A-B), ratio of IgG1-IC/IgG3-IC binding (C) and IgG1- and IgG3-IC stimulated TNFα-release (index) (D-E) in DMARD- and steroid naive early RA patients (*n* = 20) and in healthy controls (HC) (*n* = 33).

Having detected changes in monocyte FcR expression and IgG load in the early RA patients, we asked whether the RA monocyte FcγR function was affected. Although not significantly proven, we observed trends of inferior binding of IgG1-ICs (**Fig 2A**) and superior binding of IgG3-ICs in the RA cohort (**Fig 2B**) compared to the HC. Thus, when the ratios of IgG1-IC/IgG3-IC binding were compared we saw a significant difference between the groups where the RA patients displayed a lower ratio than the HC (**Fig 2C**). This suggests a possible altered IgG subclass specific IC handling via monocyte FcγRs in early RA. Consequently we further analysed the in vitro signaling capacity of the monocytes in terms of TNFα secretion in response to immobilized IgG. When stimulated with IgG1 or IgG3, the RA monocytes produced less TNFα than did HC monocytes, although statistical significance was reached only with IgG3 (**Fig 2D and 2E**). When the monocytes were stimulated with non-FcR ligands such as LPS and PMA, no difference in TNFα secretion could be observed between patients and controls (data not shown), indicating an intact TNFα production per se in early RA monocytes.

### Monocyte FcγR function reflects RA disease state

As the FcγR function was impaired in the RA cohort we considered whether this could be associated with the patients' disease activity. Indeed, a negative correlation of monocyte IC-binding with the tender and swollen joint counts and with the DAS28-CRP was observed (**Table 2**). Furthermore, the monocyte capacity of IC-mediated TNFα release was negatively correlated with patient-reported global health. Interestingly, negative correlations of the IgG1-IC binding with the IgG-RF (r = - 0.5310, p = 0.0283) and IgM-RF (r = - 0.5530, p = 0.0213) serum levels were detected (data not shown). These data suggest that disease activity in early RA is reflected by the monocytes' poor ability to deal with and signal in response to IgG-ICs, and that RA-specific auto-abs could be involved in this process.

### Changes in Ig concentrations and FcR expressions after anti-rheumatic treatment

After 3–4 months of anti-rheumatic treatment all patients were re-evaluated at a FU visit. The mean conc of CRP, Ig isotypes and all IgG subclasses had decreased significantly compared with the FV (data not shown). However, the mean CRP conc (6.3 mg/L, normal reference range 0–5 mg/L) remained slightly elevated, while the mean conc of the Igs had declined to the normal reference range. The mean values of DAS28, HAQ-score, and the VAS-scores for pain, patient global and morning stiffness were all significantly reduced at FU (data not shown).

**Fig 3:**
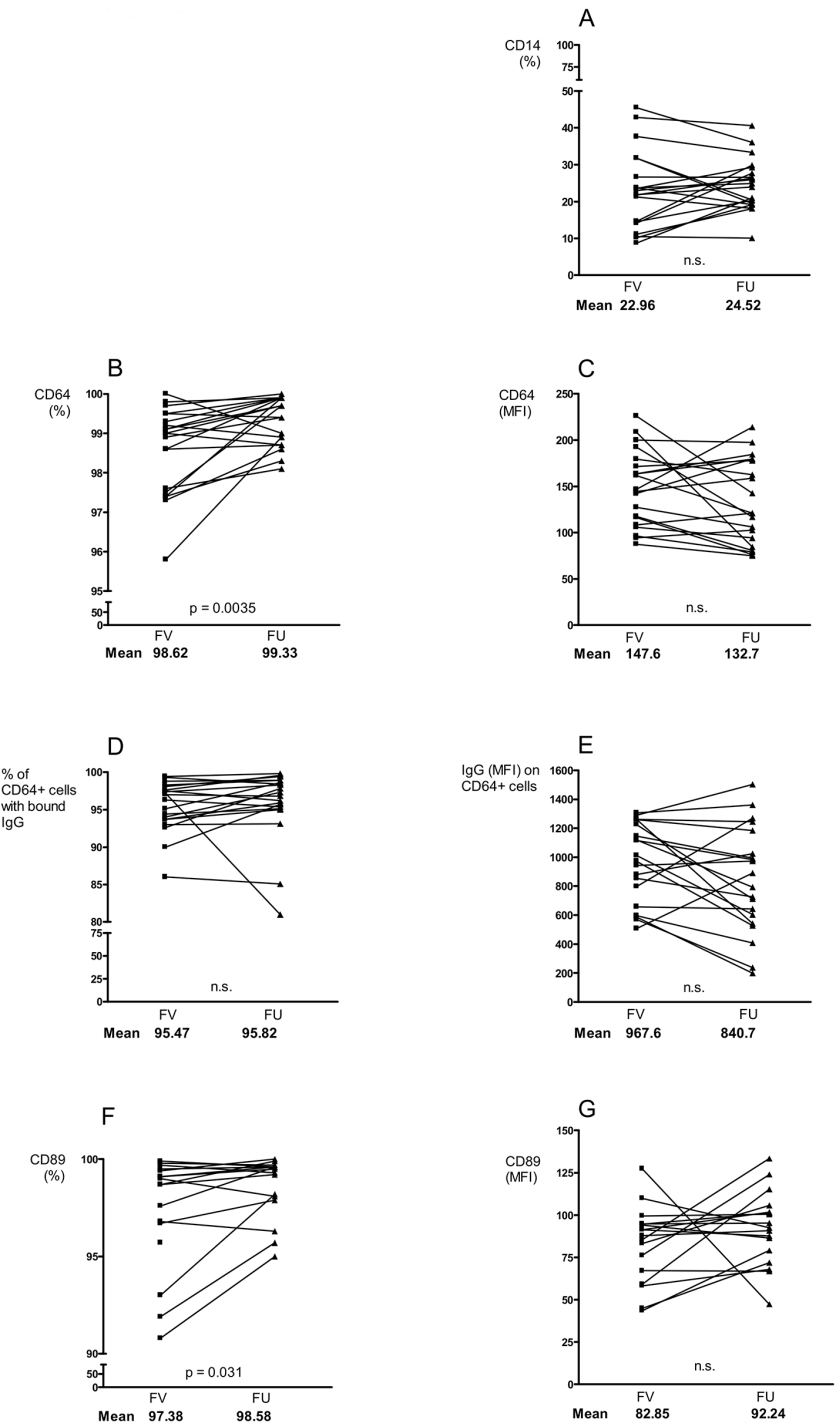
Effect of methotrexate and prednisolone treatment on monocytes and FcR receptors. Frequency and expression of CD14 (A), CD64 (B-C), IgG bound to CD64+ cells (D-E) and CD89 (F-G) on monocytes before and after 3–4 months of anti-rheumatic treatment in DMARD- and steroid naive early RA patients (*n* = 20). (FV = first visit, FU = follow up).

The number of CD14-expressing monocytes remained stable during the treatment period (**Fig 3A**). However, although the number of CD64 expressing cells increased (**Fig 3B**), we noticed a tendency of decreasing CD64 expression per cell (**Fig 3C**). Interestingly, the degree of CD64 MFI reduction was correlated with the reductions of morning stiffness (p = 0.0093) and HAQ (p = 0.0543) (data not shown). The frequency of IgG-bearing CD64⁺ monocytes was unchanged at FU (**Fig 3D**). But a tendency of lower IgG burden per CD64⁺ cell was observed (**Fig 3E**). The number of monocytes expressing CD89 had increased at FU (**Fig 3F**) and this could also be observed for the CD89 expression per cell (**Fig 3G**), although not significantly proven.

### Different treatment outcomes on FcRs and ab concentrations in good and non-responders

**Fig 4:**
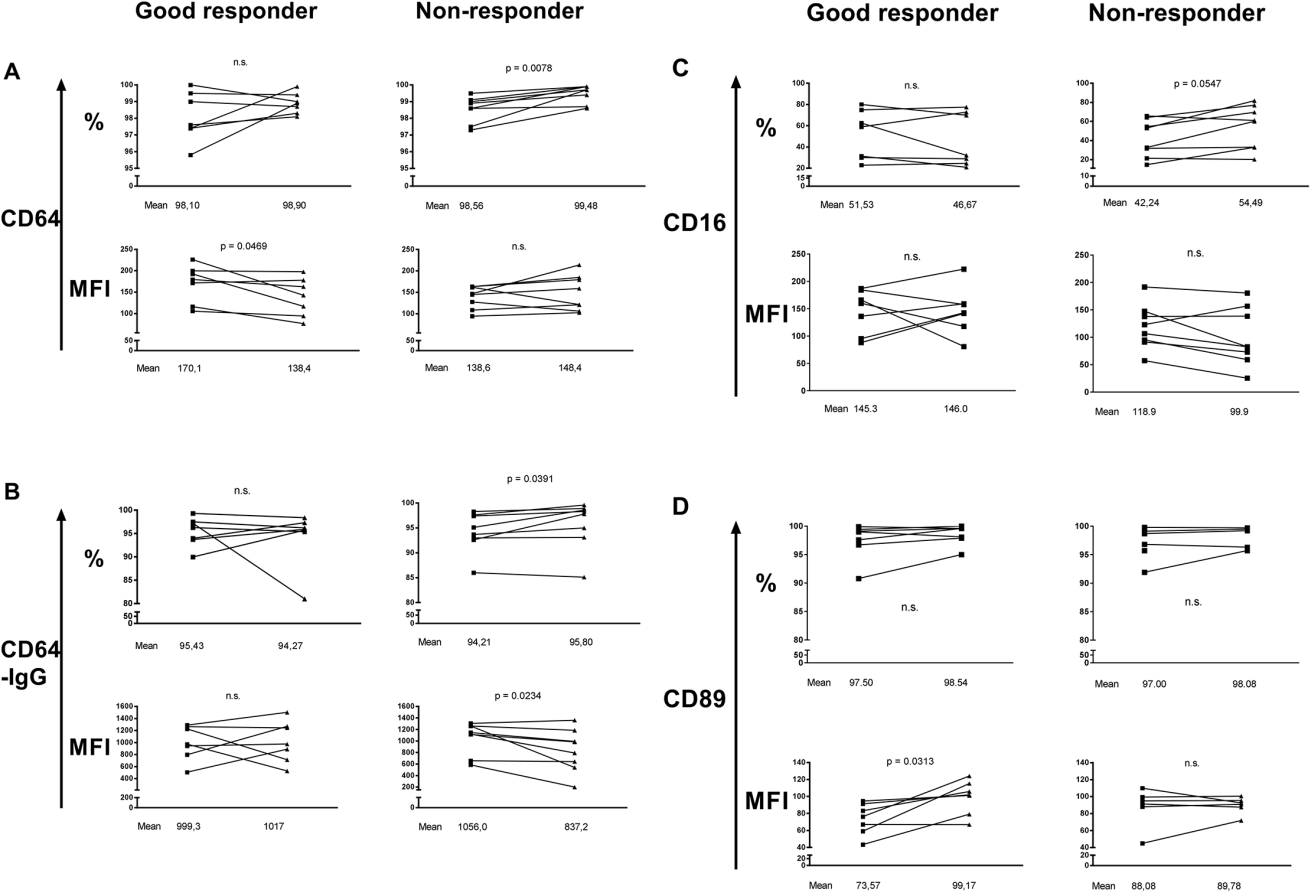
Reduction of CD64 expression after anti-rheumatic treatment in good responders. The monocyte expressions of CD64 (A), IgG bound CD64+ cells (B), CD16 (C) and CD89 (D) before and after treatment with methotrexate and prednisolone in early RA patients, defined as good responders (*n* = 7) or non responders (*n* = 8). (FV = first visit, FU = follow up, MFI = mean fluorescent intensity).

In order to learn if anti-rheumatic treatment affects the impaired FcγR activity in early RA, we then defined the patients' treatment response by applying the EULAR response criteria and compared the good responder with the non-responder data. At the FU visit the good responders excelled in having down-regulated CD64 MFI but maintained a heavy IgG load (**Fig 4A and 4B**). These effects were not observed in the non-responders who instead increased both the number of CD64^⁺^ monocytes and the number of CD64^⁺^cells with cell-bound IgG. Interestingly, the IgG load per monocyte was lighter among the non-responders following treatment (**Fig 4B**). No statistical difference was observed regarding the monocyte CD16 expressions between the responder groups (**Fig 4C**). However, in contrast to non-responders, the good responders showed increased CD89 expression at FU (**Fig 4D**).

As regards to the auto-ab levels prior to the anti-rheumatic treatment, the mean concentration of IgG-ACPA was lower in the good responders (875.1 U/L) than in the non-responders (1610.1 U/L), but no significant change in the mean ACPA levels was observed after the treatment period in either group. The mean IgM RF titres were comparable at the FV in both good and non-responders (310.4 and 312.4 IU/ml, respectively) (data not shown). However, at FU only the good responders had managed to significantly reduce their IgM RF titre (37.3 IU/L compared to 237.9 IU/ml in the non-responders).

### Changes in FcR expressions correlate with alterations of disease activity markers

Since the anti-rheumatic treatment caused various changes in monocyte FcR expressions in both good and non-responders, we then asked if these alterations could reflect changes in markers of disease activity. For good responders a small drop in CD64 MFI correlated with greater reductions in ESR and Ig levels (data not shown). Interestingly, the pre-treatment monocyte CD64 expression in the good responders was higher (MFI 170.1) than in the non-responders (MFI 138.6) (**Fig 4A**). We therefore conclude that the elevated monocyte CD64 surface expression was sufficient to deal with the only moderately increased amount of Igs (and ICs) in the good responders. We also noticed that a greater increase in CD89 MFI in the good responders at FU reflected greater improvements in the SJC and HAQ-score, and a greater reduction of serum IgG1 (data not shown). For non-responders an increase in the number of CD64⁺ cells, or CD16⁺ cells, as well as a reduction in the amount of surface-bound IgG reflected an increase in the SR, respectively (data not shown). These results could indicate that an insufficient amount of monocyte CD64 for binding IgG or ICs is unfavorable in early RA.

### After a successful treatment, good responder monocytes are in an inactivated state

**Fig 5:**
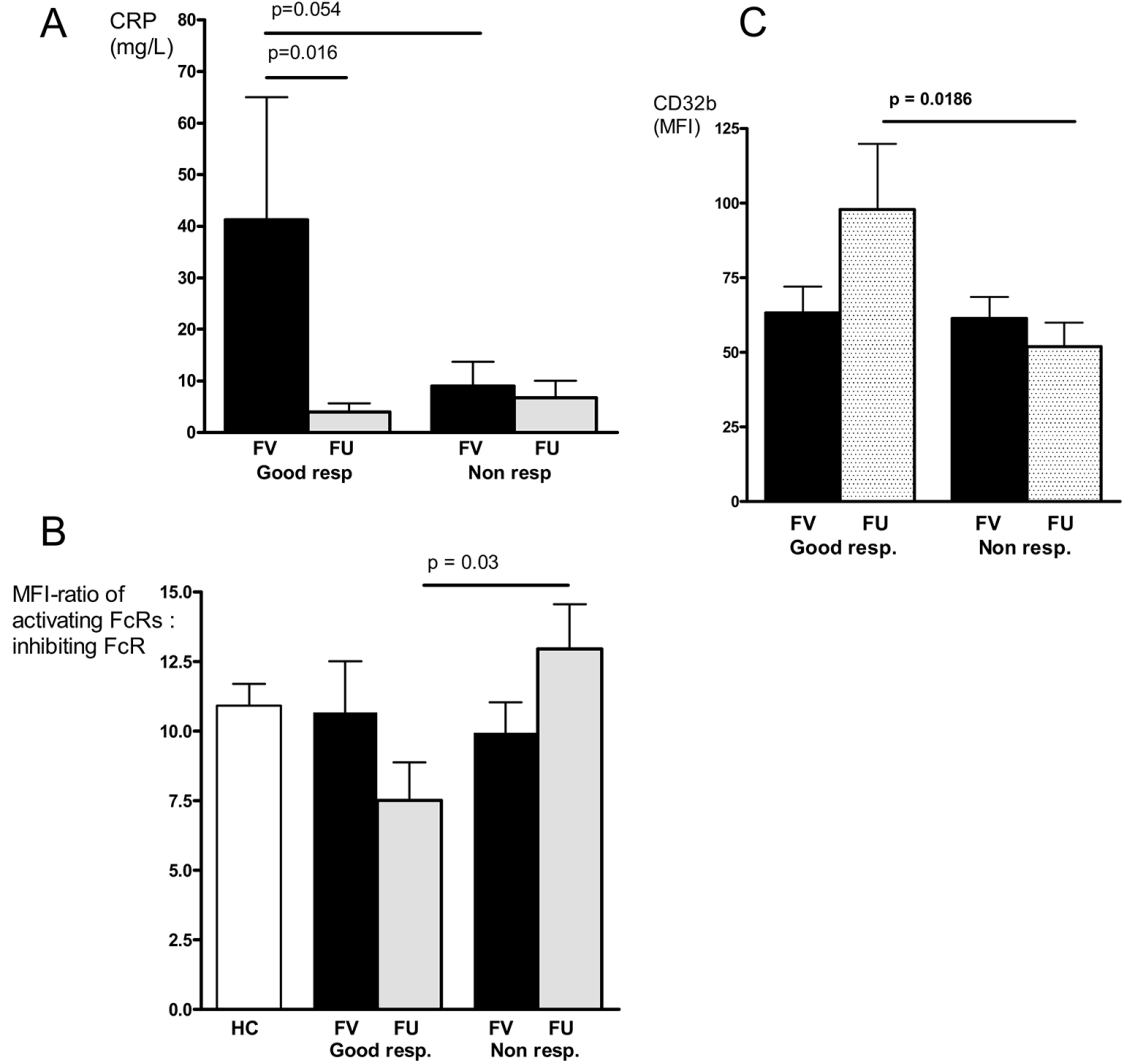
Characteristics of good and non responders in early RA. CRP-concentrations (A), the activating:inhibiting FcR expression ratio (B), and the monocyte CD32b expression (C) in good (*n* = 7) versus non responders (*n* = 8) before and after treatment with methotrexate and prednisolone in early RA. (the activating:inhibiting FcR expression ratio was defined as the ratio of the sum of the MFIs of the activating CD64 + CD32a + CD16a divided by the MFI of the inhibiting CD32b).

We further considered if there were any additional parameters that could distinguish good responders from non-responders at FU, since no differences in disease activity markers between these groups were noted at first visit (**S1 Table**). We observed a difference in the degree of reduction in the serum CRP-level between the two responder statuses; the good responders, who displayed higher pre-treatment CRP-levels, effectively reduced their CRP conc following treatment (**Fig 5A**). Unexpectedly, the non-responders presented lower mean CRP levels initially, that did not change during treatment. Other interesting observations were the decrease in the ratio of activating and inhibiting FcγRs (defined as the ratio of the sum of the MFIs of CD64, CD32a, CD16a divided by the MFI of CD32b) (**Fig 5B**), and the the up-regulation of the inhibitory CD32b in the good responders at FU (**Fig 5C**). In contrast, the non-responder monocytes maintained a low CD32b expression and had a higher ratio of activating and inhibiting FcγRs following treatment (**Fig 5B and 5C)**. These data could imply an inactivated state of RA monocytes after a successful anti-rheumatic therapy.

### Elevated sCD64 in plasma of early RA patients

**Fig 6:**
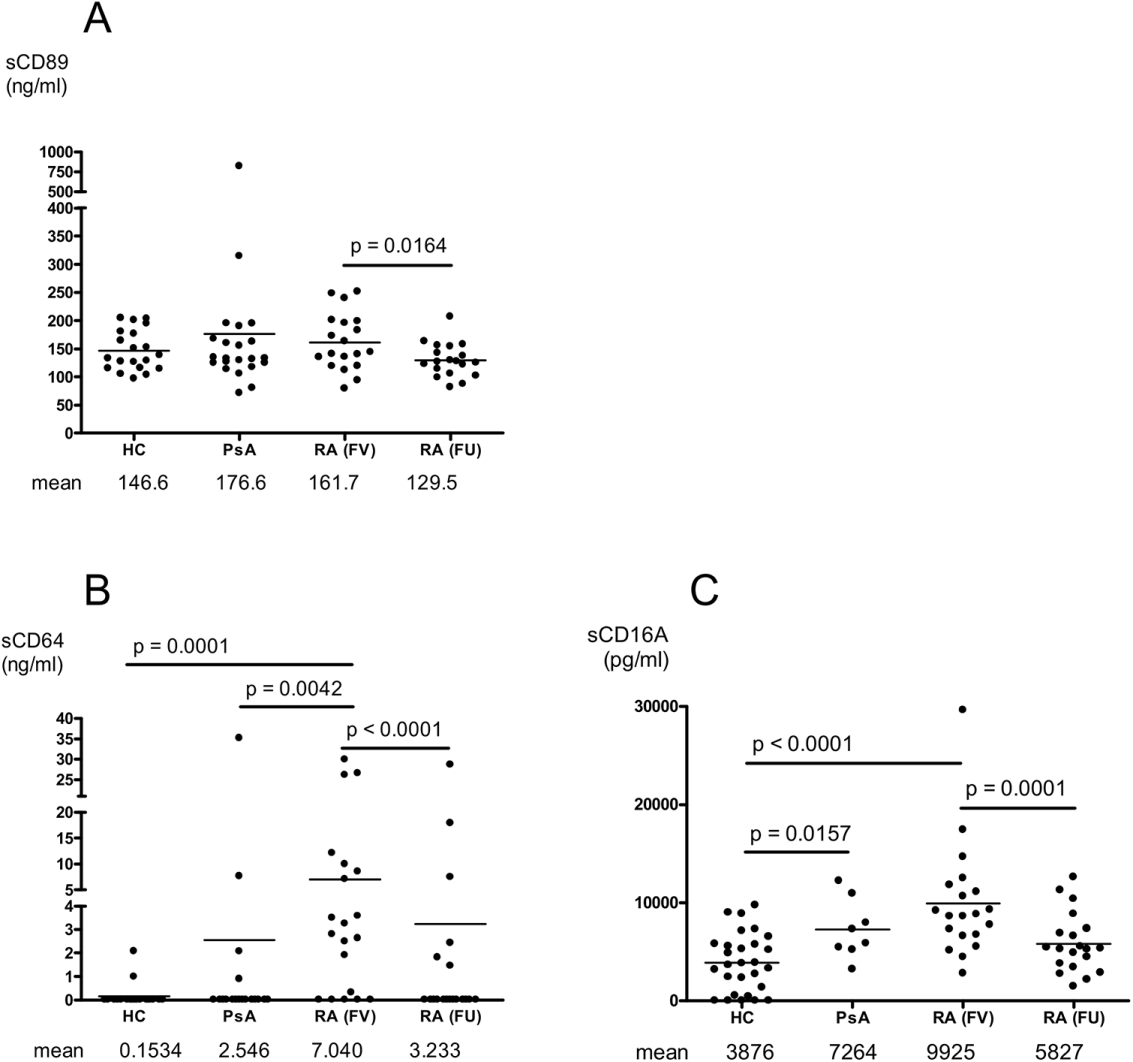
Soluble FcR levels in early RA are regulated by anti-rheumatic treatment. The plasma levels of sCD89 (A), sCD64 (B) and sCD16a (C) in healthy controls (HC)(n = 20), in patients with active polyarticular psoriatic arthritis (PsA) (n = 22), and in early RA patients (n = 19) before and after treatment with anti-rheumatics. (FV = first visit, FU = follow-up).

Since membrane FcR expressions reflect only a part of the instantaneous receptor turnover we were further interested in evaluating soluble forms of CD89, CD64 and CD16A in patients and controls in order to detect a possible protagonist among these receptors. A sex- and aged-matched population of 20 psoriatic patients with active polyarticular disease served as reference group. We found no difference in the plasma sCD89 concentrations between the patients (RA and PsA, respectively) and controls (**Fig 6A**), but the levels decreased in the RA patients after the anti-rheumatic treatment. A marked elevation of sCD64 was evident in the early RA patients (**Fig 6B**). This was not observed in the HC nor in the PsA reference group. The mean sCD64 levels in the early RA patients correlated with the monocyte CD64 expression (MFI) and the amount of IgG bound to CD64⁺ cells (**Table 2**). After treatment the RA patients had significantly reduced the sCD64 levels (**Fig 6B**). These data suggest a specific role of CD64 in early RA.

Regarding sCD16A, which constitutes the soluble form of the monocyte-specific FcγRIIIa, we observed an increase in both patient populations in contrast to the HC (**Fig 6C**), indicating a less specific reaction of an inflammatory state. Indeed, significant correlations of sCD16A with serological, patient reported and doctor observed signs of joint inflammation were observed in the RA patients (**Table 2**). Following anti-rheumatic treatment, the RA patients had significantly reduced the sCD16A in plasma (**Fig 6C**).

## Discussion

With this study we want to draw the attention to CD64 as an important player in early autoantibody positive RA. In our analyses no other membrane-bound FcγRs were found significantly modified, and sCD64 was elevated only in RA plasma. The early naïve RA monocytes were characterized by up-regulation of CD64 and membrane bound IgG. The cytokine environment in RA certainly contribute to this; IFNγ and G-CSF have been shown to stimulate monocyte IgG binding and/or CD64 expression [48, 49]. In RA, G-CSF and IFNγ are elevated in sera of symptom-free “pre-RA”-individuals, in patients with established disease, and in early and late RA synovial fluid [50, 51]. In situations of severe systemic inflammation such as septicemia, the monocytic CD64 expression increases due to the effects of these cytokines. However, this is restored during recovery of inflammation [52]. This monocyte CD64 up-regulation may influence the susceptibility to ICs, the ability for antigen presentation, and further monocyte differentiation [53–56]. In our study, we were unable to detect changes in the monocyte CD64 expression in the non-responders despite adequate treatment. We therefore conclude that maintaining a high monocyte CD64 expression characterizes a state of persistent arthritis.

The high IgG load we noticed on the RA monocytes implies a state of constant FcγR occupancy. A similar finding is seen in long standing RA [42]. Since CD64 was the sole significantly up-regulated FcγR in the patients, this high affinity receptor would appear to be responsible for this IgG burden. In addition, CD64 presents greater binding affinity for IgG1 than IgG3. Both of these subclasses were increased in the RA serum, IgG1 to a greater extent than IgG3. We therefore suspect that CD64 is most likely occupied by IgG1 in early RA. Since IgM RF is raised in RA serum in active disease [57] and has high binding specificity for IgG1 and IgG3, a high monocytic IgG load is a possible prerequisite for IgM RF-ICs to interact with CD64. On the other hand, IgG3 binding to CD16 is 3-fold stronger than IgG1. This could facilitate pathogenic IgG3-ICs to further activate RA monocytes via low affinity receptors [58].

We believe that the observed FcγR occupancy in the RA monocytes contributes to the decrease in the TNFα production. This effect is probably due to a negative impact on the intracellular signaling pathways downstream of the FcγR, as otherwise stimulated monocytic TNFα production was similar in patients and controls. Interestingly, states of acute inflammation such as pancreatitis and cardiac surgery demonstrate a similar monocyte anergy with reduced TNFα production, and ICs may inhibit in vitro-activated human monocytes by suppressing intracellular pathways via CD64 [59–61]. These data suggest that high levels of CD64 and monocyte-bound IgG may promote monocyte suppression, as seen in the good responders in our study. Furthermore, the altered monocyte FcγR functions we observed reflected the RA disease activity; an inferior IC binding was followed by more joint symptoms. Interestingly, we noticed that the IC binding capacity of the non-responder monocytes at the FV was inferior to that of the good responders (p = 0.03, data not shown). This suggests that optimal IC handling is of importance to prevent RA maintenance, and this also raises the question whether monocytic IC binding capacity could predict the outcome in RA patients treated with methotrexate and steroids. However, the observed patients were too few in this study to draw definite conclusions.

Clinical improvement at FU was mainly correlated with changes in monocyte CD64 and CD89 expressions. What seems to facilitate remission in the good responders was a generally eased pressure on monocytic FcRs. Indeed, the conc of all 3 soluble forms decreased at FU. For this, the reduced Ig levels after treatment or the effect of the drugs may have been responsible [23, 35, 62, 63]. An increase in CD89 MFI was also accompanied by symptom relief and a reduction of sCD89. Since monocyte CD89 expression correlates negatively with serum IgA levels in health and disease, we assume that with a favorable response to treatment, a decrease in the IgA-ICs binding to CD89 will stop receptor shedding into the circulation [64–66]. On the other hand, CD89 expression is dependent on the FcRγ chain expression [67]. One could hypothesize that a favorable treatment outcome (i. e. reduced CD64 expression in the good responders) results in an increased availability of free FcRγ chains which per se would allow an increase in membrane-anchored CD89. Moreover, the significant correlations of sCD16A with several laboratory, patient reported and doctor observed signs of joint inflammation in our study stand out. We therefore propose sCD16A as an independent marker of RA disease activity.

Considering CRP the good and non-responders differed regarding their pre-treatment serum levels, although disease activity was comparable between the groups (**S1 Table**). Consistent with a previous infliximab-study in established RA, our data showed a poorer outcome in low pre-treatment CRP individuals [68]. Indeed, the non responders maintained low CRP levels during the treatment course. Since CRP interacts with FcRs for immune response modulation [69] this is an interesting observation, especially as most studies in established RA report CRP as a sole negative prognostic factor. However, animal studies provide evidence of a protective role of CRP in early arthritis, and CRP-bound antigens in vitro have no pro-inflammatory potential, compared with IgG-ICs [70–72]. These data call for further studies on the role of CRP in RA.

In conclusion, we emphasise the role of CD64 in the early phase of autoantibody positive RA where up-regulation of membrane-bound and soluble CD64 appears to be pathognomonic. Monocyte CD64 down-regulation should be taken into consideration as an objective factor of good treatment response. We also propose sCD64 as an RA specific biomarker, but further and larger studies on this topic are warranted, including seronegative patients.

## Supporting Information

S1 Fig CD64 is up regulated on all PBMC subsets. The expression of CD64 on gated PBMCs defined by their CD16 expression level (high, low or negative) in healthy controls (HC) and early RA patients before and after treatment with methotrexate and prednisolone (A-C) and percentages thereof (D-E).
(TIF)

S1 Table Baseline characteristics of patients with newly diagnosed rheumatoid arthritis who experience good or no response to a combination treatment with methotrexate and steroids. Presented are mean values at the first visit.
(DOCX)
