# Supplementary material for: Elevated Membrane and Soluble CD64: A Novel Marker Reflecting Altered FcγR Function and Disease in Early Rheumatoid Arthritis That Can Be Regulated by Anti-Rheumatic Treatment
Source: PLoS One. 2015 Sep 25;10(9):e0137474. doi: 10.1371/journal.pone.0137474 (PMC4583189; doi:10.1371/journal.pone.0137474)
Supplement: S1 Table — Presented are mean values at the first visit. (DOCX) [file pone.0137474.s002.docx]

| **Parameter** | **Good responders (n=7)** | **Non-responders (n=8)** | **p- value** |
| --- | --- | --- | --- |
| Swollen joint count (n) | 7.4 | 5.3 | 0.28 |
| Tender joint count (n) | 7.9 | 8.3 | 0.88 |
| HAQ | 1.13 | 0.6 | 0.07 |
| Pain (mm) | 70.4 | 46.3 | 0.03 |
| Patient global (mm) | 69.3 | 32.3 | 0.01 |
| Morning stiffness (mm) | 69 | 53.2 | 0.13 |
| ESR (mm/h) | 35 | 19.4 | 0.14 |

**Supplement Table 1** Baseline characteristics of patients with newly diagnosed rheumatoid arthritis who experience good or no response to a combination treatment with methotrexate and steroids. Presented are mean values at the first visit.
